# Association between serum levels of insulin-like growth factor-binding proteins at admission and outcomes at 3 months after acute ischemic stroke

**DOI:** 10.1080/07853890.2025.2472867

**Published:** 2025-03-06

**Authors:** Yuyi Zhu, Huan Wang, Ting Cui, Mingxi Chen, Yaqi Chen, Simiao Wu, Zilong Hao, Shihong Zhang, Xinyi Leng, Deren Wang

**Affiliations:** a Department of Neurology, West China Hospital, Sichuan University, Chengdu, China; b Center of Cerebrovascular Diseases, West China Hospital, Sichuan University, Chengdu, China; c Department of Medicine and Therapeutics, The Chinese University of Hong Kong, Hong Kong SAR, China

**Keywords:** Ischemic stroke, insulin-like growth factor-binding proteins, biomarker, prognosis

## Abstract

**Background:**

Insulin-like growth factor-binding proteins (IGFBPs) contribute to central nervous system development and may influence recovery after stroke. This study aimed to determine whether serum IGFBPs levels in acute ischemic stroke (AIS) patients are associated with 3-month outcomes.

**Materials and methods:**

We retrospectively reviewed data from AIS patients admitted within 24 h after stroke onset, and who had been prospectively enrolled in the Chengdu Stroke Registry. Serum IGFBPs 4, 6 and 7 levels at admission were compared between patients experienced good outcome (modified Rankin Scale scores of 0-2) or poor outcome (scores of 3-6) at 3 months after stroke onset. Factors associated with good outcome were identified using logistic regression.

**Results:**

Among 194 patients, 115 (59.3%) experienced good outcome at 3 months. Patients with good outcome showed significantly higher levels of all three IGFBPs at admission. Good outcome was independently associated with higher serum levels of IGFBP 4 (OR 1.013, 95% CI 1.005-1.020) and IGFBP 7 (OR 1.012, 95% CI 1.003-1.021) after adjustment for potential confounders. Adding either or both IGFBPs to a model based on conventional clinical factors significantly improved good outcome prediction, with net reclassification improvement of 41.9-54.5% and integrated discrimination improvement of 3.8-5.8%. The model containing both IGFBPs predicted good outcome with an area of 0.878 (95% CI 0.827-0.929) under the receiver operating characteristic curve.

**Conclusions:**

Higher serum IGFBPs 4 and 7 levels may be associated with greater probability of good outcome at 3 months after AIS.

## Introduction

Ischemic stroke, which accounts for more than 60% of all strokes, is a major cause of death and disability worldwide [1,2]. Accurately predicting the prognosis of stroke patients can help individualize treatment and management, ensuring adequate monitoring and timely intervention for those likely to need it most. While several predictive models have been developed based on diverse clinical and demographic variables [3–8], their accuracy varies widely [9]. Incorporating the levels of certain blood markers into these models can improve their performance [10–12].

We wondered whether adding serum levels of insulin-like growth factor-binding proteins (IGFBPs) might improve prognostic models. These proteins can function independently of insulin-like growth factor to promote cell proliferation and angiogenesis, while protecting against apoptosis [13,14]. Administering exogenous IGFBP 4 to a rat model of ischemic stroke can reduce infarct volume, while administering it to neuronal cultures can protect them from injury induced by oxygen deprivation [15]. IGFBP 6 has been shown to protect neurons from injury [16]. IGFBP 7 appears to be crucial for proper development and maintenance of the blood-brain barrier [17,18], and its upregulation after stroke may be important for driving angiogenesis in the brain during recovery [19].

These observations imply that serum levels of IGFBPs at admission may help predict recovery after acute ischemic stroke (AIS). Meanwhile, elevated levels of IGFBP 6 in blood at admission have been linked to higher possibility of early neurologic improvement after stroke [20]. Whether levels of IGFBPs in serum at admission can help predict prognosis of AIS patients is unclear; also unclear is whether lower or higher levels of the proteins are associated with better prognosis.

Thus, the present study retrospectively examined AIS patients in order to evaluate potential associations of serum levels of IGFBPs 4, 6 and 7 with 3-month outcomes.

## Materials and methods

This study was conducted in accordance with the 2013 revision of the Declaration of Helsinki, and reported in accordance with ‘Strengthening the reporting of observational studies in epidemiology’ (STROBE) guidelines [21].

### Patients

Medical data were retrospectively analyzed for a consecutive series of patients with first-ever or recurrent stroke who had been admitted to the Department of Neurology at West China Hospital between December 2018 and December 2021 and who, at admission, had been prospectively enrolled into the Chengdu Stroke Registry [22,23]. At the time of enrollment, patients provided written informed consent for their anonymized medical data to be analyzed and published for research purposes.

Patients were enrolled in the present study if they were at least 18 years old at admission, were diagnosed with AIS based on computed tomography or magnetic resonance imaging of the brain, and were admitted within 24 h of symptom onset. Detailed inclusion and exclusion criteria of the patients in this study have been reported in a previous analysis [23].

### Clinical assessment of patients

Data at admission were extracted from the Registry about demographic characteristics, clinical features, vascular risk factors, reperfusion therapy, stroke severity (assessed using the National Institutes of Health Stroke Scale), and TOAST classification, which was defined as previously described [23]. Patients were classified as experiencing a good outcome if their score on the modified Rankin Scale was 0-2 [24]; ­otherwise, they were classified as experiencing a poor outcome. The outcome was determined based on follow-up telephone interviews with patients or their caregivers as described previously [23].

### Assay of IGFBPs in serum at admission

Peripheral blood was sampled within 48 h after arrival in the emergency department, and serum was isolated and stored immediately at −80 °C. For the present study, samples were thawed and assayed in commercially available enzyme-linked immunosorbent assays (Jiangsu Meibiao Biotechnology, Nanjing, Jiangsu, China) for IGFBP 4 (catalog no. MB-0067A), IGFBP 6 (catalog no. MB-0064A) and IGFBP 7 (catalog no. MB-4339A). Assays were performed off-site at K J Biotechnology (Chengdu, China) according to the instructions of the assay manufacturer. Technicians performing the assays were blinded to the characteristics and outcomes of patients.

### Statistical analysis

Data were analyzed statistically using IBM SPSS Statistics for Windows 27.0 (IBM, Armonk, NY, USA), GraphPad Prism 9.5 (GraphPad Software, San Diego, CA, USA), and R for Windows 4.2.2. Results associated with a two-tailed *p* < 0.05 were considered statistically significant.

Continuous data were reported as median (interquartile range, IQR) because they showed a skewed distribution based on the Shapiro-Wilk test. Categorical data were reported as n (%). Associations between good outcome and variables were assessed as odds ratios (ORs) and 95% confidence intervals (95% CIs) in univariate logistic models with good outcome as the dependent variable.

Potential independent associations of IGFBPs levels with good outcome were identified through multivariable binary logistic regression that adjusted for all variables that were associated with good outcome in univariate logistic regression analysis at a significance level of *p* < 0.10. Regression results were reported in terms of adjusted odds ratios (ORs) and 95% confidence intervals (CIs). Potential associations of IGFBPs levels with good outcome were further explored through multivariable-adjusted restricted cubic spline analysis, which adjusted for variables that were significant in univariate logistic regression analysis at a significance level of *p* = 0.10. Knots were specified at the 5th, 35th, 65th, and 95th percentiles [25]. In addition, we performed subgroup analyses to evaluate the association between IGFBPs and good outcomes, adjusting for the covariates mentioned earlier. Interaction terms were included in the models, and the likelihood ratio test was used to assess potential effect modifiers.

We assessed the ability of different models to predict good outcome in our cohort. The ‘conventional’ model contained all variables, except IGFBP levels, that were associated with good outcome in univariate analysis at a significance level of *p* < 0.10. Other models also contained the serum level of either IGFBP 4 or 7, or the serum levels of both IGFBPs. The predictive performance of the models containing IGFBP(s) was compared to that of the conventional model in terms of net reclassification improvement, integrated discrimination improvement, and area under the receiver operating characteristic curve (AUC). AUCs for different models were compared using the DeLong test.

## Results

The enrolled 194 patients were predominantly male (63.9%) and had a median age of 66 years (IQR 55-72 years). The median NIHSS score on admission was 8 (IQR 3-14), and median IGFBPs levels in serum on admission were 230.64 ng/mL (IQR 202.44-263.28 ng/mL) for IGFBP 4, 8520.40 ng/mL (IQR 7575.40-9980.50 ng/mL) for IGFBP 6, and 218.42 ng/mL (IQR 196.95-240.49 ng/mL) for IGFBP 7. A total of 115 patients (59.3%) showed good outcome at 3 months after stroke onset.

Univariate binary logistic regression analyses showed that patients with good outcome had significantly lower median age (61 vs 69 years, *p* < 0.001) and median NIHSS score at admission (4 vs 14, *p* < 0.001), but they were significantly more likely to be drinkers (35.7% vs 21.5%, *p* = 0.035) or current smokers (48.7% vs 32.9%, *p* = 0.029; Table 1). Good outcome was associated with a higher frequency of strokes due to small-artery occlusion (23.5% vs 2.5%, *p* = 0.002) and lower frequency of concurrent atrial fibrillation (20.0% vs 45.6%, *p* < 0.001; Table 1).

**Table 1.** Univariate binary logistic regression analyses assessing the prediction of good outcome.

Characteristic	Odds Ratio	95% Confidence Interval	p *
Age, yr	0.943	0.916-0.970	<0.001*
Male	1.658	0.915-3.005	0.096
Current smoking	1.935	1.067-3.508	0.029*
Alcohol assumption	2.021	1.051-3.903	0.035*
Time from onset until admission, h	0.991	0.951-1.033	0.673
Systolic blood pressure, mmHg	0.996	0.986-1.006	0.393
Diastolic blood pressure, mmHg	0.999	0.984-1.014	0.905
NIHSS score	0.812	0.764-0.863	<0.001*
Vascular risk factors			
Hypertension	0.918	0.510-1.653	0.776
Diabetes mellitus	0.914	0.480-1.742	0.786
Hyperlipidemia	0.650	0.266-1.588	0.342
Coronary artery disease	1.244	0. 509-3.038	0.632
Atrial fibrillation	0.299	0.158-0.564	<0.001*
Stroke history	0.462	0.177-1.207	0.109
Reperfusion therapy	0.596	0.334-1.063	0.079
Stroke etiology			0.005*
Large-artery atherosclerosis	1.000 (reference)	NA	
Cardioembolism	0.511	0.282-1.076	0.081
Small-artery occlusion	10.530	2.359-47.009	0.002*
Other determined cause	1.170	0.186-7.349	0.867
Undetermined cause	4.290	0.898-20.492	0.068
Levels of IGFBPs in serum, ng/ml			
IGFBP 4	1.009	1.004-1.015	0.002*
IGFBP 6	1.000	0.999-1.000	0.028*
IGFBP 7	1.010	1.003-1.017	0.004*

*p < 0.05 (univariate binary logistic regression). IGFBP, insulin-like growth factor-binding protein; NA: not applicable; NIHSS: National Institutes of Health Stroke Scale.

**Figure 1.**
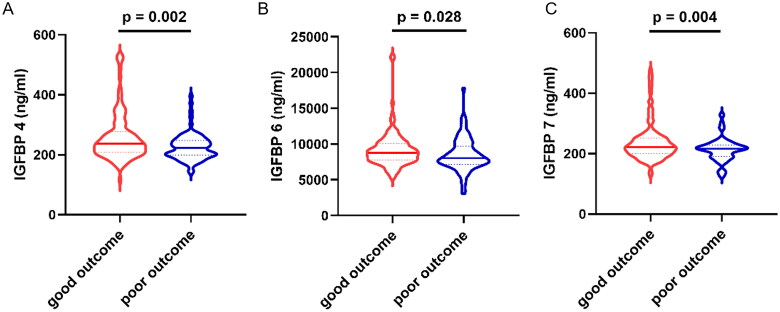
Comparison of levels of (A) IGFBP 4, (B) IGFBP 6 and (C) IGFBP 7 in serum at admission between patients who experienced good or poor outcome at 3 months after stroke onset. The violin plots indicate the median and interquartile range; dotted lines indicate the 10th and 90th percentiles. P values are shown from univariate binary logistic regression. IGFBP: insulin-like growth factor-binding protein.

**Figure 2.**
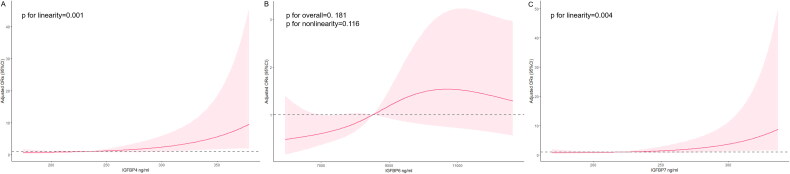
Restricted cubic spline analysis of potential relationships between good outcome and levels of (A) IGFBP 4, (B) IGFBP 6 or (C) IGFBP 7 in serum at admission. All analyses adjusted for the following variables: age, sex, current smoking, alcohol assumption, NIHSS score at admission, atrial fibrillation, stroke etiology, and reperfusion therapy. In each panel, the red line indicates adjusted OR, the pink shading shows 95% confidence intervals, and the horizontal dotted line indicates OR = 1. OR: odds ratio; CI: confidence interval; IGFBP: insulin-like growth factor-binding protein.

Patients with good outcome showed significantly higher median levels of all three IGFBPs at admission (Figure 1): IGFBP 4, 238.47 vs 222.57 ng/mL (*p* = 0.002); IGFBP 6, 8752.50 vs 8030.40 ng/mL (*p* = 0.028); and IGFBP 7, 222.09 vs 215.80 ng/mL (*p* = 0.004). After adjustment for numerous potential confounders, independent associations with good outcome were observed for IGFBP 4 (OR 1.013, 95% CI 1.005-1.020, *p* = 0.001) and IGFBP 7 (OR 1.012, 95% CI 1.003-1.021, *p* = 0.007) but not IGFBP 6 (OR 1.000, 95% CI 0.999-1.000, *p* = 0.252; Table 2). In multivariable-adjusted restricted cubic spline analysis, good outcome showed a positive dose-response relationship with serum levels of IGFBPs 4 and 7 but not 6 (Figure 2).

**Table 2.** Multivariable binary logistic regression to identify factors independently associated with good outcome.*

Factor	OR (95%CI)	p	OR (95%CI)	p	OR (95%CI)	P
Age	0.961 (0.927-0.996)	0.028	0.959 (0.926-0.994)	0.022	0.964 (0.931-0.999)	0.044
Sex	0.786 (0.282-2.188)	0.644	0.874 (0.326-2.345)	0.790	0.856 (0.314-2.333)	0.762
Current smoking	1.405 (0.420-4.703)	0.581	1.212 (0.391-3.754)	0.739	1.185 (0.384-3.656)	0.768
Alcohol assumption	1.203 (0.346-4.178)	0.771	1.425 (0.446-4.554)	0.551	1.356 (0.420-4.380)	0.611
NIHSS score at admission	0.804 (0.738-0.876)	<0.001	0.819 (0.757-0.885)	<0.001	0.810 (0.747-0.878)	<0.001
Atrial fibrillation	0.790 (0.116-5.380)	0.809	0.660 (0.112-3.890)	0.647	0.600 (0.094-3.832)	0.589
**Stroke etiology**						
Large-artery atherosclerosis	Ref		Ref		Ref	
Cardioembolism	1.207 (0.184-7.992)	0.844	1.389 (0.242-7.962)	0.713	1.571 (0.255-9.657)	0.626
Small-artery occlusion	3.690 (0.705-19.301)	0.122	3.803 (0.749-19.320)	0.107	3.524 (0.688-18.043)	0.131
Other determined cause	0.419 (0.023-7.683)	0.558	0.600 (0.041-8.828)	0.709	1.602 (0.107-23.960)	0.733
Undetermined cause	2.082 (0.376-11.531)	0.401	1.765 (0.322-9.693)	0.512	1.944 (0.355-10.645)	0.433
Reperfusion therapy	1.895 (0.750-4.792)	0.177	2.158 (0.891-5.227)	0.088	1.990 (0.811-4.884)	0.133
Levels of IGFBP in serum						
IGFBP 4	1.013 (1.005-1.020)	0.001	–		–	
IGFBP 6	–		1.000 (0.999-1.000)	0.252	–	
IGFBP 7	–		–		1.012 (1.003-1.021)	0.007

* All analyses adjusted for the following variables: age, sex, current smoking, alcohol assumption, NIHSS score at admission, atrial fibrillation, stroke etiology, and reperfusion therapy.

CI: confidence interval; IGFBP: insulin-like growth factor-binding protein; NIHSS: National Institutes of Health Stroke Scale; OR: odds ratio; Ref: reference.

Subgroup analyses revealed a significant interaction between serum IGFBP 4 levels and reperfusion therapy (p for interaction = 0.017, Figure S1). However, no significant interactions were observed between IGFBP 4 or 7 and other potential effect modifiers (all p for interaction > 0.05). Higher serum IGFBP 4 and 7 levels were significantly associated with good outcome across most subgroups (Figures S1 and S2).

A model involving only conventional clinical factors predicted good outcome in our cohort with an AUC of 0.853, which improved significantly to 0.878 (*p* = 0.038) after serum levels of both IGFBPs 4 and 7 were added to the model, but not after serum levels of only one or the other IGFBP were added (Table 3). Relative to the model with only conventional clinical factors, a model that also included serum levels of IGFBPs, whether only one or both, showed significantly better predictive power in terms of net reclassification improvement and integrated discrimination improvement.

**Table 3.** Comparison of the ability of different models to predict good outcome in the patients in the study.*

Model *	AUC (95%CI)	p	NRI (95%CI)	p	IDI (95%CI)	p
Conventional	0.853 (0.797-0.908)	Ref	Ref	–	Ref	–
Conventional + IGFBP 4	0.874 (0.822-0.927)	0.103	59.7 (33.5-85.9)	<0.001	5.3 (2.4-8.3)	<0.001
Conventional + IGFBP 7	0.868 (0.815-0.920)	0.144	41.9 (14.6-69.2)	0.003	3.8 (1.2-6.4)	0.004
Conventional + IGFBPs 4 and 7	0.878 (0.827-0.929)	0.038	54.5 (28.3-80.8)	<0.001	5.8 (2.7-9.0)	<0.001

* The conventional model included age, sex, current smoking, alcohol assumption, NIHSS score at admission, atrial fibrillation, stroke etiology, and reperfusion therapy.

AUC: area under the receiver operating characteristic curve; CI: confidence interval; IGFBP: insulin-like growth factor-binding protein; IDI: integrated ­discrimination improvement; NRI: net reclassification improvement; Ref: reference.

## Discussion

Our retrospective analysis of data from a rigorously curated stroke registry provides evidence linking higher levels of IGFBPs 4 or 7 in serum at admission to greater probability of good outcome at 3 months after AIS. Restricted cubic spline analysis revealed positive, linear dose-response relationships between IGFBP 4 and IGFBP 7 levels and improved outcomes. Furthermore, incorporating these biomarkers into conventional clinical models significantly enhanced predictive performance, as demonstrated by improved AUC, NRI, and IDI metrics. These findings lay the groundwork for future studies to elucidate the role of IGFBPs 4 and 7 in AIS recovery and assess their potential as prognostic biomarkers.

Our findings suggest that IGFBP 4 may play a beneficial role in stroke recovery, consistent with its protective effects observed in related contexts [15,26,27]. For example, exogenous IGFBP 4 has been shown to reduce infarct volume in a rat model of ischemic stroke and to inhibit the production of apoptotic proteins in neuronal cultures exposed to oxygen-glucose deprivation [15]. Similarly, IGFBP 4 protected cardiac tissue in mice from ischemic injury by limiting DNA damage and inhibiting Wnt/β-catenin signaling [26]. Additionally, the combination of IGFBP 4 and vascular endothelial growth factor promoted angiogenesis after myocardial infarction in mice [27]. Likewise, IGFBP 7 has demonstrated protective effects in tissue injury, including upregulation of anti-oxidant enzymes, attenuation of oxidative stress damage [28], and reduction of inflammatory responses [29–31]. It has also been shown to promote angiogenesis and preserve integrity of the blood-brain barrier [18,32]. Our findings are consistent with these observations and provide further evidence supporting the involvement of IGFBPs in stroke recovery. However, further experimental research is needed to confirm these mechanisms and explore the therapeutic potential of IGFBPs in the context of stroke.

Our findings reveal associations between higher IGFBP 4 and IGFBP 7 levels and better outcomes, which appear to contrast with prior studies. Higher IGFBP 4 levels have been linked to increased risk of acute stroke [33], mortality, and major adverse cardiac events in myocardial infarction patients [34,35], as well as greater risk of coronary artery disease and B-type coronary lesions in stable cardiovascular disease populations [36]. Similarly, higher IGFBP 7 levels have been associated with increased risk of coronary artery disease [37]. In contrast, we observed no association between IGFBP 6 levels and good outcomes, differing from prior reports suggesting a link between higher IGFBP 6 levels and early neurologic recovery after stroke [20]. These discrepancies may be attributed to differences in patient populations (ischemic heart disease vs. stroke), stroke severity, comorbidities, treatment strategies, or the limited sample size in our study, which may have reduced statistical power to detect independent associations. Further research is essential to clarify the roles of IGFBPs 4, 6, and 7 in ischemic injury across the heart and brain and to validate their potential role in stroke outcomes.

Our findings should be interpreted with caution due to several limitations. First, IGFBPs levels were measured only once, limiting our ability to assess their dynamic changes over time. Future studies with repeated measurements during hospitalization and follow-up are needed to clarify their role in recovery and prognostic utility. Second, despite controlling for multiple confounders, residual confounding cannot be completely ruled out, underscoring the need for prospective cohort studies. Third, the retrospective design may have introduced selection bias, as some serum samples were unavailable due to their prior use in other research projects. This limited the sample size, potentially affecting the generalizability of our findings. Larger, multi-ethnic studies are essential to validate and extend our findings.

Nonetheless, our study is the first to explore the relationship between IGFBPs and stroke outcomes, providing preliminary evidence for their potential role in stroke recovery. Further research is required to confirm these findings and elucidate the underlying mechanisms in diverse populations and clinical settings.

## Conclusion

Higher levels of IGFBPs 4 and 7 in serum at admission may be associated with higher chance of achieving a good outcome at 3 months after AIS. The two biomarkers significantly increased the predictive power of conventional clinical factors alone in predicting good outcomes. Future research should clarify whether and how the two IGFBPs promote recovery in the ischemic brain.

## Supplementary Material

Supplemental Material

## Data Availability

The data that support the findings of this study are available from the corresponding author, Deren Wang, upon reasonable request.
