# Supplementary material for: Association between serum levels of insulin-like growth factor-binding proteins at admission and outcomes at 3 months after acute ischemic stroke
Source: Ann Med. 2025 Mar 6;57(1):2472867. doi: 10.1080/07853890.2025.2472867 (PMC11892070; doi:10.1080/07853890.2025.2472867)

**Supplementary Figure 1.** Subgroup analysis of association between IGFBP 4 level and good outcome in acute ischemic stroke patients. The presented ORs and 95% CIs are based on binary logistic regression analyses with adjustment for age, sex, current smoking, alcohol assumption, NIHSS score at admission, atrial fibrillation, stroke etiology, and reperfusion therapy, except for the stratified variable. Abbreviations: IGFBP, insulin-like growth factor-binding protein; LAA, Large-artery atherosclerosis; CE, Cardioembolism; OR, odds ratio; CI, Confidence interval


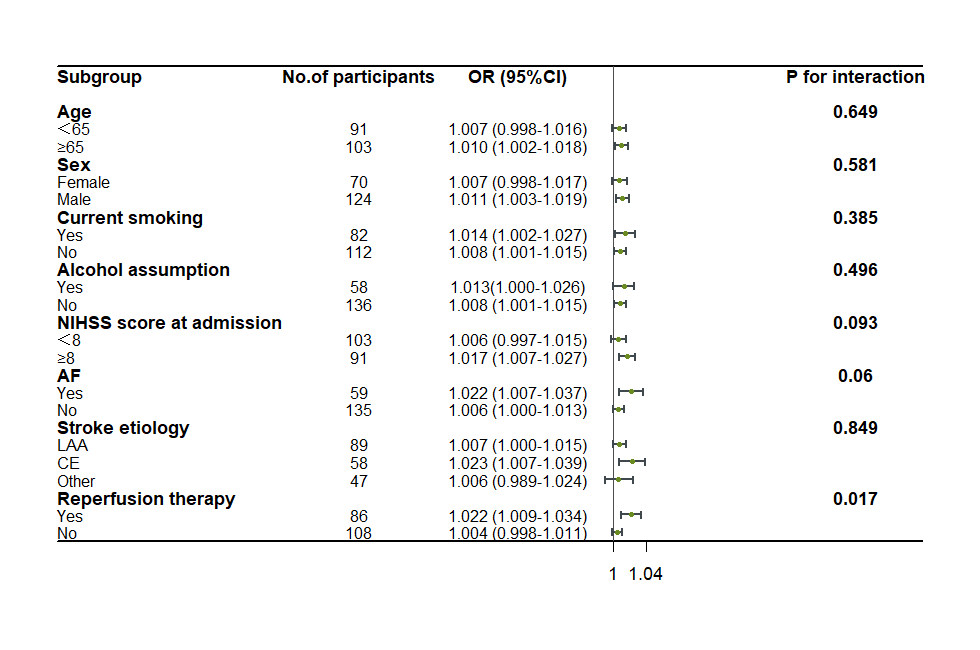


**Supplementary Figure 2.** Subgroup analysis of association between IGFBP 7 level and good outcome in acute ischemic stroke patients. The presented ORs and 95% CIs are based on binary logistic regression analyses with adjustment for age, sex, current smoking, alcohol assumption, NIHSS score at admission, atrial fibrillation, stroke etiology, and reperfusion therapy, except for the stratified variable. Abbreviations: IGFBP, insulin-like growth factor-binding protein; LAA, Large-artery atherosclerosis; CE, Cardioembolism; OR, odds ratio; CI, Confidence interval.


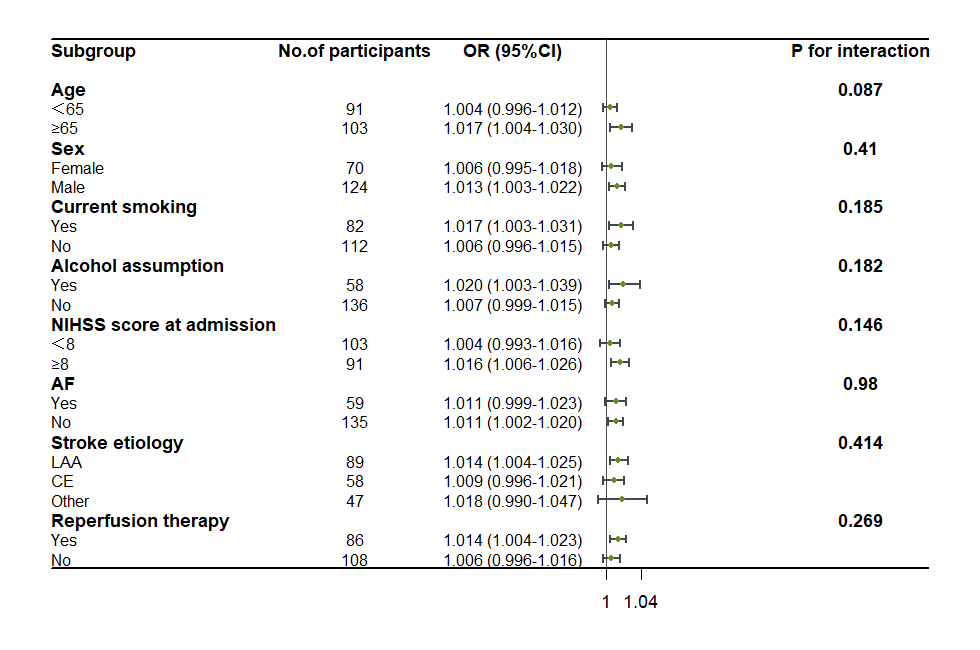

Supplement: Supplemental Material [file IANN_A_2472867_SM3862.docx]
